# Isoform-specific deletion of PKM2 constrains tumor initiation in a mouse model of soft tissue sarcoma

**DOI:** 10.1186/s40170-018-0179-2

**Published:** 2018-05-31

**Authors:** Talya L. Dayton, Vasilena Gocheva, Kathryn M. Miller, Arjun Bhutkar, Caroline A. Lewis, Roderick T. Bronson, Matthew G. Vander Heiden, Tyler Jacks

**Affiliations:** 1 David H. Koch Institute for Integrative Cancer Research and Department of Biology, Massachusetts Institute of Technology, Cambridge, MA 02139 USA; 2 Rodent Histopathology Core, Harvard Medical School, Boston, MA 02111 USA; 3 Department of Medical Oncology, Dana-Farber Cancer Institute, Boston, MA 02115 USA; 4 Howard Hughes Medical Institute, Massachusetts Institute of Technology, Cambridge, MA 02139 USA

**Keywords:** PKM2, Soft tissue sarcoma, Cancer

## Abstract

**Background:**

Alternative splicing of the *Pkm* gene product generates the PKM1 and PKM2 isoforms of the glycolytic enzyme pyruvate kinase. PKM2 expression is associated with embryogenesis, tissue regeneration, and cancer. PKM2 is also the pyruvate kinase isoform expressed in most wild-type adult tissues, with PKM1 restricted primarily to skeletal muscle, heart, and brain. To interrogate the functional requirement for PKM2 during tumor initiation in an autochthonous mouse model for soft tissue sarcoma (STS), we used a conditional *Pkm2* allele (*Pkm2*^*fl*^) to abolish PKM2 expression.

**Results:**

*PKM2* deletion slowed tumor onset but did not abrogate eventual tumor outgrowth. *PKM2*-null sarcoma cells expressed PKM1 with tumors containing a high number of infiltrating PKM2 expressing stromal cells. End-stage *PKM2*-null tumors showed increased proliferation compared to tumors with a wild-type *Pkm2* allele, and tumor metabolite analysis revealed metabolic changes associated with PKM2 loss.

**Conclusions:**

While PKM2 is not required for soft tissue sarcoma growth, PKM2 expression may facilitate initiation of this tumor type. Because these data differ from what has been observed in other cancer models where PKM2 has been deleted, they argue that the consequences of *PKM2* loss during tumor initiation are dependent on the tumor type.

**Electronic supplementary material:**

The online version of this article (10.1186/s40170-018-0179-2) contains supplementary material, which is available to authorized users.

## Background

Proliferating cells, including cancer cells, favor aerobic glycolysis and display increased glucose consumption and lactate production compared to many non-proliferating cells. Expression of the M2 isoform of pyruvate kinase (PKM2), the glycolytic enzyme that catalyzes the last step in glycolysis, has been implicated in promoting this metabolic state of cancer cells.

The pyruvate kinase gene, *Pkm*, encodes the PKM1 and PKM2 isoforms, which are the result of alternative splicing of mutually exclusive exons. The PKM1 isoform includes exon 9 but not exon 10 and shows a pattern of expression that is restricted in the adult largely to the heart, brain, and skeletal muscle. PKM2, on the other hand, includes exon 10 but not exon 9. Expression of the PKM2 isoform is widespread in adult epithelial tissues and has been associated with embryogenesis, tissue regeneration, and cancer [1, 2]. Importantly, expression of PKM2 impacts the fate of glucose, in part through regulated enzymatic activity. While PKM1 is a constitutively active enzyme, PKM2 enzymatic activity can be inhibited by a variety of cellular signaling events, many of which are associated with proliferation [3–7]. Consistent with this notion, activation of PKM2 enzymatic activity in cancer cells through either exogenous expression of PKM1 or use of small molecule activators leads to delayed xenograft tumor formation [8, 9].

PKM2 is highly expressed in many tumor types, and a number of metabolic and non-metabolic functions in cancer have been attributed to PKM2 [1, 5, 8, 10–15]. However, whether PKM2 is required for tumor formation remains a controversial question. Recent studies of PKM2 function in autochthonous mouse models of cancer have revealed a complicated picture of PKM2 function in cancer. For example, loss of *PKM2* in a leukemia and breast cancer model led to contrasting results: while *PKM2* loss limited progression in one case, it led to accelerated tumor growth in the other [16, 17]. PKM2 deletion also accelerated tumor growth in a mouse medulloblastoma model [18]. Loss of *PKM2* in a colon cancer model driven by APC loss did not alter tumor initiation, growth, or progression [19]. Finally, germline loss of PKM2 led to late onset of hepatocellular carcinoma, at least in part through non-cell autonomous means [2]. In no cases was PKM2 absolutely required for tumor formation; however, the different response to *PKM2* loss in various tumors might be indicative of distinct tissue metabolic requirements.

In this study, we used a conditional *Pkm2* allele (*M2*^*fl*^) to assess the requirement for PKM2 in a mouse model of soft tissue sarcoma. In this model, Cre-mediated activation of oncogenic Kras and loss of *p53* (*KP*) in the hind limb of mice leads to the formation of soft tissue sarcomas within 3 months [20]. Loss of *PKM2* in this context led to delayed tumor initiation and decreased tumor penetrance. The tumors that formed in *KP M2*^*fl/fl*^ mice expressed PKM1 instead of PKM2. Metabolite analysis of *KP M2*^*−/−*^ sarcoma tissue showed changes in the pool sizes of several metabolites, arguing that metabolism in these tumors changes as a result of PKM2 deletion and consequent PKM1 expression.

## Methods

### Mouse strains and treatments

All animal work was conducted in accordance with a protocol approved by the MIT Committee on Animal Care. KP and *M2* flox mouse strains have been described previously [17, 21]. All animals were maintained on a mixed C57BL/6J × 129SvJ genetic background.

Sarcoma tumorigenesis was initiated as previously described by intramuscular infection of mice with Ad5-CMV-Cre (University of Iowa Gene Transfer Core) [20, 22]. For histologic analysis, tissues were fixed in 4% formalin, embedded in paraffin, and sections were stained with hematoxylin and eosin (H&E).

### Immunofluorescence

For immunofluorescence analysis, mice were perfused with PBS, followed by perfusion with 2% paraformaldehyde (PFA). After an overnight fixation in 4% PFA at 4 **°**C, tissues were placed in 30% sucrose overnight at 4 **°**C with shaking. Tissue was allowed to equilibrate in OCT:PBS (1:1) solution and embedded in OCT (Tissue-Tek). Frozen sections (10 μm thick) were cut on a CryoStar NX70 (Thermo Scientific). PKM2 antibody (1:500, CST 4053) was used.

### Immunohistochemistry (IHC)

IHC was performed on 4-μm-thick sections using ABC Vectastain kit (Vector Labs) with antibodies to PKM1 (1:500, CST 7067), PKM2 (1:500, CST 4053), and phospho-Histone H3 (1:200, CST 9701). The staining was visualized with DAB (Vector Labs, SK-4100), and the slides were counterstained with hematoxylin. For dual staining, DAB and ImmPACT VIP (Vector Labs, SK-4605) were used. Hematoxylin and eosin (H&E) staining was performed on a Varistain Gemini automated slide stainer (Thermo Shandon). Soft tissue sarcoma human tissue array, SO2081, was purchased from US Biomax Inc. (Rockville, MD USA). The TMA was scored for PKM1 and PKM2 intensity. Tumors that showed no positive staining were given a score of 0, those with weak staining were given a score of 1, those with strong positive staining were given a score of 2, and those with very strong staining were given a score of 3.

ImageJ software was used for quantification of pHH3 staining, and four to five 10× fields were counted per tumor.

### RNA isolation and qRT-PCR

RNA was isolated from flash frozen crushed sarcoma tissue following manufacturer’s instructions for Trizol (Invitrogen). One to 2 μg of RNA was reverse transcribed following manufacturer’s instructions for High Capacity cDNA Reverse Transcription Kit (Applied Biosystems). KAPA SYBR Fast mix (Kapa Biosystems, KK4604) was used for quantitative PCR with primers listed below. Expression levels were calculated relative to β-actin and normalized to WT samples.

**Table:** 

Target gene	Forward	Reverse
*-actin*	GGCATAGAGGTCTTTACGGATGTC	TATTGGCAACGAGCGGTTCC
*Pkm*	TGACACCTTCCTGGAACACA	TTCAGCATCTCCACAGATCG
*Pkm1*	GTCTGGAGAAACAGCCAAGG	TCTTCAAACAGCAGACGGTG
*Pkm2*	GTCTGGAGAAACAGCCAAGG	CGGAGTTCCTCGAATAGCTG

### Immunoblotting

Sarcoma tissue lysates were generated from flash frozen ground whole tumors following lysis with ice cold RIPA buffer, supplemented with HALT phosphatase and protease inhibitors (Thermo-Scientific, PI-78420 and 87786). The following antibodies were used for immunoblotting: anti-Hsp90 (1:1000; CST 4877), anti-PKM1 (1:5000; Cell Signaling, 7067), and anti-PKM2 (1:5000; Cell signaling, 4053).

#### Metabolite extractions and LC/MS analysis

Metabolites were extracted from ground flash frozen whole tumors by methanol extraction. For polar metabolite analysis, dried metabolite samples were resuspended in 100 mL of water, centrifuged at 15,000×*g* at 4° C for 10 min, and 1 mL of supernatant was injected for LC/MS analysis using QExactive Orbitrap mass spectrometer as previously described [23, 24].

#### EdU incorporation

Mice were given one pulse of intraperitoneally injected EdU, and tissue was harvested after 3 h. EdU was visualized using Click-iT EDU AlexFluor-647 kit for IF (Invitrogen C10340). For EdU incorporation in cell lines, cells were grown on a monolayer and treated with EdU for 1 h.

#### M2/M1 expression-associated survival analyses in human sarcoma patients

RNA-seq gene expression profiles (*n* = 265) and relevant clinical data (*n* = 261) for sarcoma (SARC) patients were obtained from the Cancer Genome Atlas data portal (TCGA; cancergenome.nih.gov/). Patients with primary tumor samples and associated survival data (*n* = 259) were ranked by the ratio of the RPKM (reads per Kb per million mapped reads) values of PKM2 and PKM1 isoform-specific exons. Values greater than one indicate higher expression of the M2 isoform compared to the M1 isoform, with higher values indicating greater M2 isoform expression. Kaplan–Meier survival analysis was conducted to compare patients with high M2/M1 expression ratio (top 20th percentile) to those in the bottom 20th percentile (*n* = 51 within each group). The log-rank test was used to assess significance. Cox proportional hazards analysis was conducted across all patients in the TCGA SARC cohort (*n* = 261) to assess the prognostic significance of the M2/M1 expression ratio. Univariate Cox regression analysis for individual characteristics was performed to benchmark single-variable hazard ratios. Subsequently, a multivariable model was implemented to estimate the prognostic value of the M2/M1 ratio while controlling for other patient characteristics (age, gender). Hazard ratio proportionality assumptions for the Cox regression model fit were validated by testing for all interactions simultaneously (*p* = 0.0856). Interaction between significant covariates (M2/M1 ratio and age) was tested using a likelihood ratio test (LRT) to contrast a model consisting of both covariates with another model consisting of both covariates and an interaction term. All survival analyses were conducted within a 5-year survival timeframe using the survival package in R (www.r-project.org).

## Results

### PKM2 is expressed in mouse and human sarcomas

**Fig. 1:**
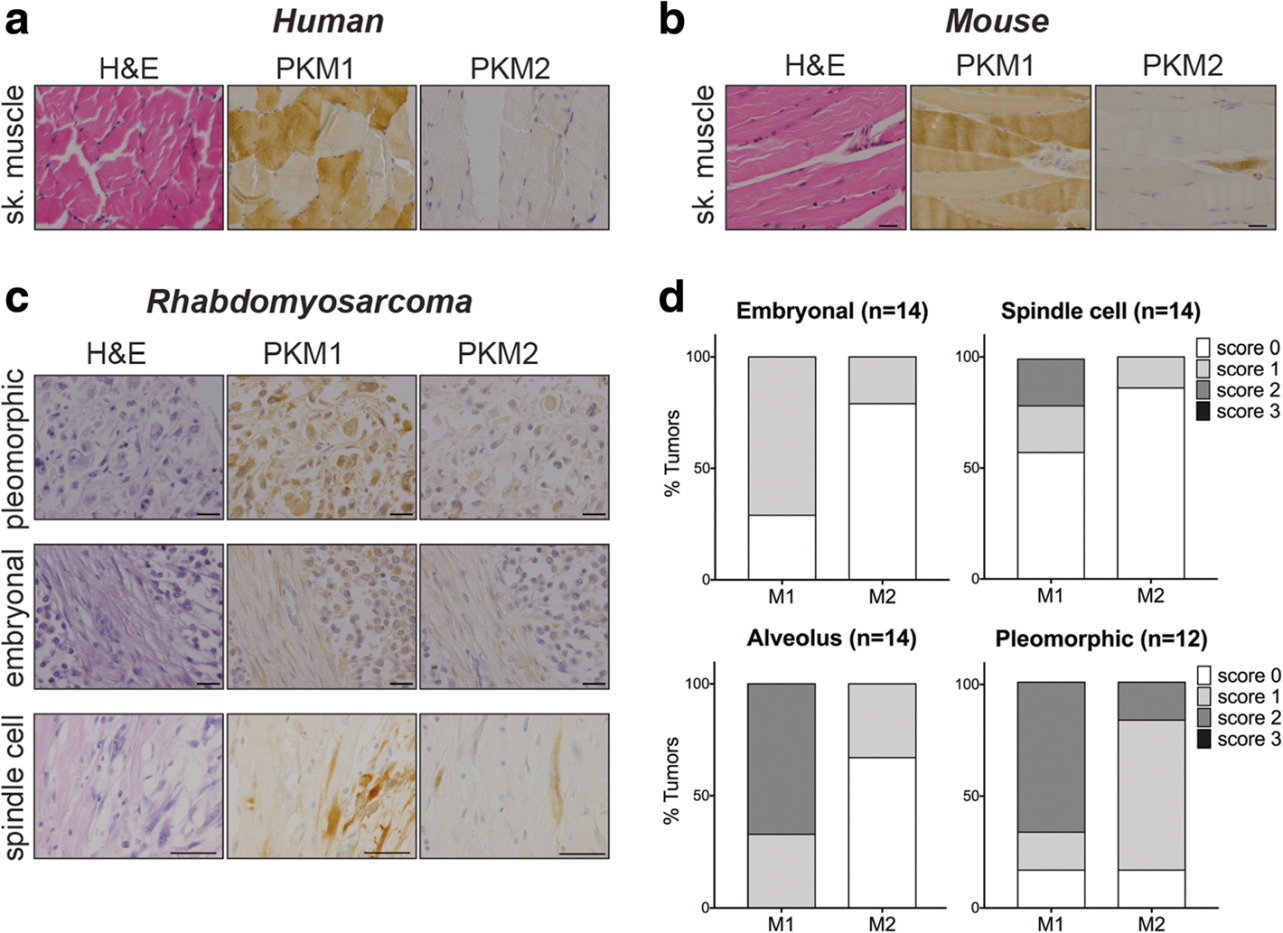
Human and mouse soft tissue sarcomas express PKM2. Representative images of IHC for PKM1 and PKM2 in human (**a**) and mouse (**b**) skeletal muscle and human rhabdomyosarcoma (**c**). Corresponding H&E images are shown. Scale bars, 20 μm. **d** Quantification of PKM1 and PKM2 staining intensities in human rhabdomyosarcoma tumors relative to rhabdomyosarcoma subtype (embryonal, spindle cells, alveolus, pleomorphic). Score 0 = no staining, score 1 = weak, score 2 = positive, or score 3 = strong

To inform our study of PKM isoform requirements in soft tissue sarcomas (STS), we examined the expression patterns of PKM1 and PKM2 in normal adult skeletal muscle and STS tissue. This was assessed by immunohistochemistry (IHC) staining using antibodies specific to either PKM1 or PKM2. IHC staining of both mouse and human skeletal muscle confirmed previous reports that PKM1 is the primary isoform expressed in this tissue (Fig. 1a, b, Additional file 1: Figure S1A) [2]. We next stained 48 primary human rhabdomyosarcomas including embryonal, spindle cell, alveolus, and pleomorphic rhabdomyosarcomas (undifferentiated sarcomas) for PKM1 and PKM2 (Fig. 1c). Consistent with the PKM expression pattern of their tissue of origin, the majority of the examined rhabdomyosarcomas showed positive staining for PKM1 (32/48, 67%). Less than half of the examined rhabdomyosarcomas showed positive staining for PKM2, arguing against an absolute requirement for PKM2 expression in this tumor type (Additional file 1: Figure S1B). PKM1 and PKM2 IHC intensity scoring revealed heterogeneity in the distribution of PKM2-expressing tumors according to rhabdomyosarcoma subtypes (Fig. 1d). In contrast to the other three examined subtypes, pleomorphic rhabdomyosarcomas contained a high percentage of PKM2-positive tumors (10/12, 83%). The majority of these tumors also expressed PKM1 (10/12, 83%).

**Fig. 2:**
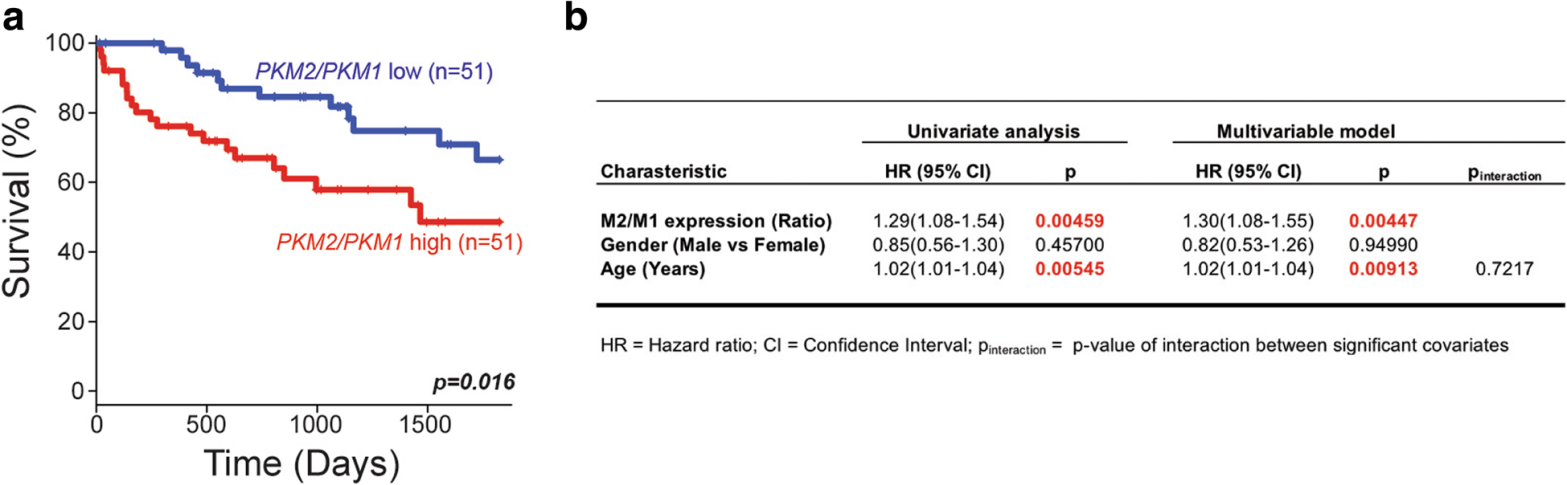
A high PKM2/PKM1 expression ratio predicts poor prognosis. **a** Kaplan–Meier 5-year survival analysis comparing patients in the top quintile of PKM2/PKM1 expression ratio (*n* = 51; red) and those in the bottom quintile (*n* = 51; blue). Log-rank test *p* value is shown. **b** Results of univariate and multivariable Cox proportional hazards model on overall survival in the TCGA sarcoma cohort (all patients). Increasing PKM2/PKM1 expression ratio shows a significant association with poor survival after controlling for other characteristics

In addition to our IHC analysis of PKM1 and PKM2 expression in primary human sarcomas, we also analyzed human sarcoma gene expression data from The Cancer Genome Atlas (TCGA) to determine whether the expression ratio of *PKM2* to *PKM1* could predict patient outcome [25]. Across > 200 sarcoma patients within TCGA, those within the highest quintile for *PKM2/PKM1* expression ratio had a significantly shorter median 5-year survival when compared to either those within the lowest quintile for *PKM2/PKM1* expression ratio, or when compared with all other patients combined (Fig. 2a, Additional file 2: Figure S2). Furthermore, Cox regression analysis using this TCGA sarcoma patient cohort showed that a high *PKM2/PKM1* ratio was independently prognostic of shorter survival when controlling for age and gender (Fig. 2b).

### *PKM2* loss constrains sarcoma tumor initiation

We were intrigued by the disparate PKM expression patterns in normal and transformed skeletal muscle tissue. While normal skeletal muscle exclusively expressed PKM1, a sizeable number of human primary tumors expressed both PKM1 and PKM2. This is in contrast to breast cancer, leukemia, and colon cancer where the normal and tumor tissue both express PKM2 [16, 17, 19]. This, in addition to the prognostic value of *PKM2/PKM1* revealed by our analysis of the TCGA sarcoma cohort, led us to postulate that expression of PKM2 may play a role in malignant transformation of skeletal muscle tissue to form STS.

To directly test this hypothesis in a well-defined genetic context, we used a genetically engineered mouse model of soft tissue sarcoma. Conditional activation of oncogenic *Kras* and inactivation of *p53* in a *Kras*^*LSL-G12D/+*^; *p53*^*fl/fl*^ (*KP*) mouse model by viral delivery of Cre recombinase to skeletal muscle tissue initiates the development of high-grade sarcomas with myofibroblastic differentiation [20]. Cross-species gene expression analysis has shown that tumors from the *KP* model closely resemble human undifferentiated pleomorphic sarcomas [26] and our IHC analysis of human pleomorphic sarcomas showed that this subtype of STS is enriched for PKM2-expressing tumors (Fig. 1d). Importantly, IHC for PKM1 and PKM2 on STS derived from the *KP* model showed that most cells in all of the examined tumors expressed both PKM1 and PKM2 (Additional file 1: Figure S1C).

**Fig. 3:**
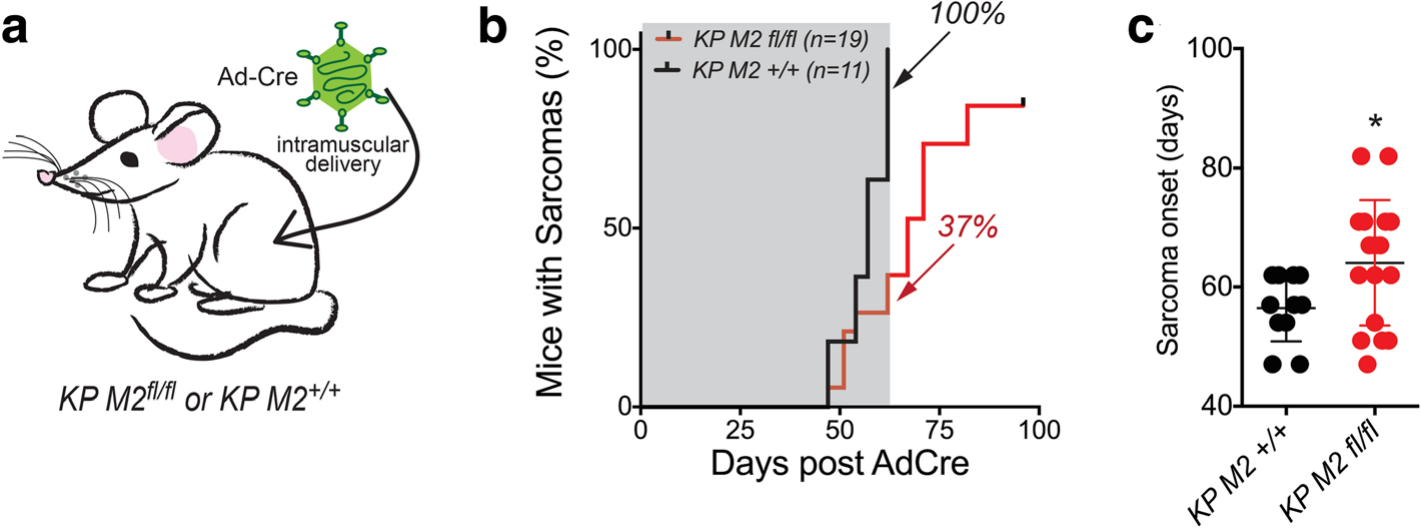
PKM2 loss delays *KP* soft tissue sarcoma initiation. KP M2^+/+^ or KP M2^fl/fl^ mice were injected intramuscularly with Ad5-CMV-Cre (**a**) and the onset of palpable sarcomas was monitored, *p* value by log-rank test is 0.003 (**b**). The percentage of total mice (*n*) with sarcomas by 62 days (gray box) is indicated. **c** Time for palpable tumor formation, *p* value using unpaired *t* test is 0.04

To investigate the role of PKM2 in sarcoma development in the *KP* model, we crossed mice with a conditional allele of *Pkm2* (*M2*^*fl*^) [17] to *KP* mice and performed intramuscular infections of cohorts of *KP M2*^*fl/fl*^ and *KP M2*^*+/+*^ mice with Ad-Cre (Fig. 3a). In contrast to similar experiments performed in other autochthonous mouse cancer models, we found that deletion of *PKM2* concomitant with sarcoma initiation delayed tumor onset and decreased penetrance of the model (Fig. 3b) (*p* = 0.003 by log-rank test). Deletion of *PKM2* delayed the date of tumor onset by approximately 8 days (56.45 days for *KP M2*^*+/+*^ versus 64.05 days for *KP M2*^*flfl*^) (Fig. 3c).

**Fig. 4:**
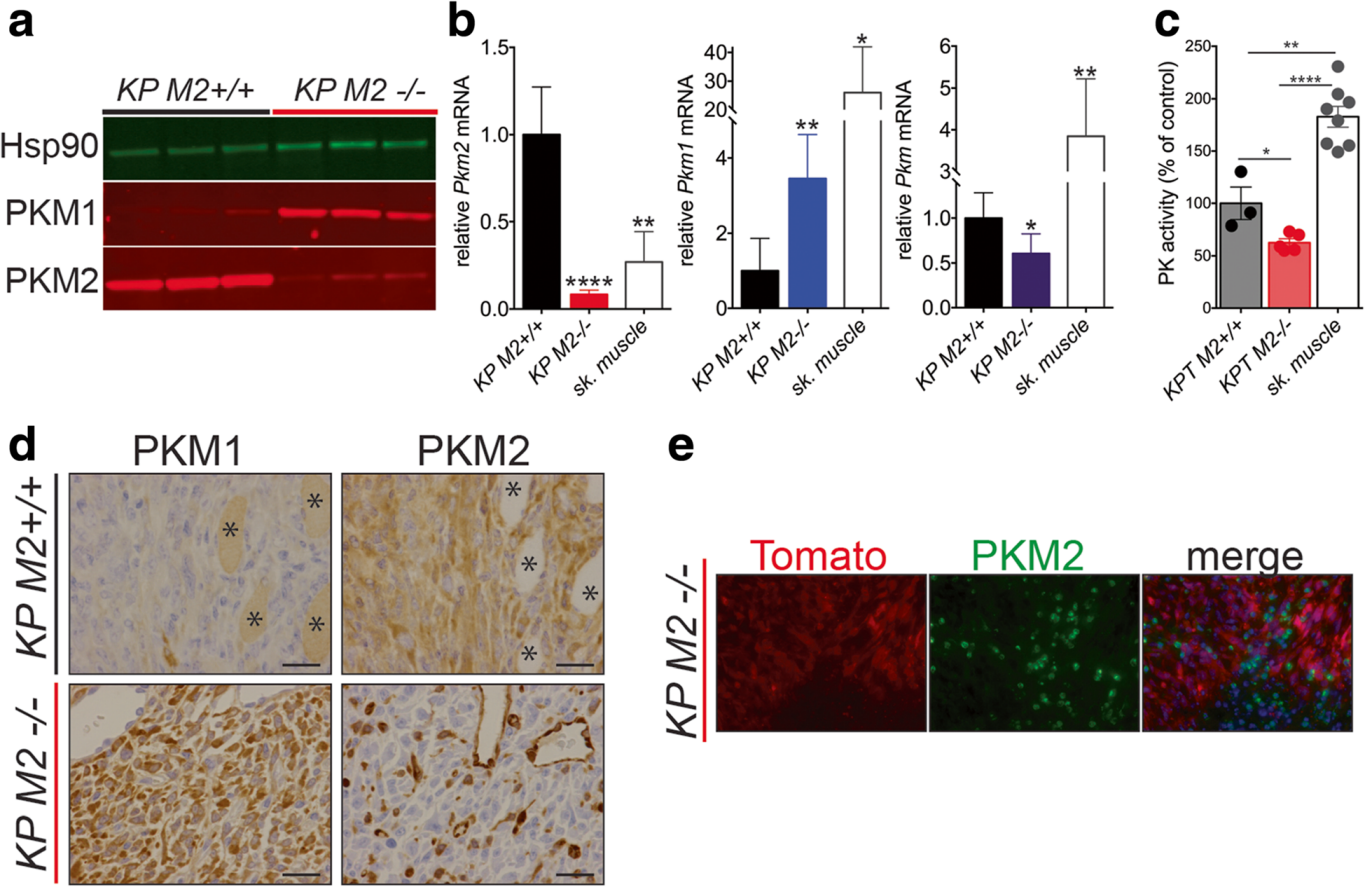
*PKM2*-deficient sarcoma tumor cells express PKM1. **a** Western blot analysis for PKM1 and PKM2 on lysates from *KP M2*^*+/+*^ or *KP M2*^*−/−*^ sarcoma tissue. Hsp90 was used as a loading control. **b** Expression of *Pkm1*, *Pkm2*, and total *Pkm* mRNA in *KP M2*^*+/+*^, *KP M2*^*−/−*^, or wild-type skeletal muscle tissue was assessed by qRT-PCR. Mean values ± SEM and *p* values using unpaired *t* test are shown, *p < 0.05; **p < 0.01; ****p < 0.0001. **c** Pyruvate kinase enzymatic activity of *KP M2*^*+/+*^, *KP M2*^*−/−*^, or wild-type skeletal muscle tissue, *p* value using unpaired *t* test is shown, *p < 0.05. **d** Representative images for PKM1 and PKM2 IHC in *KP M2*^*+/+*^ and *KP M2*^*−/−*^ sarcoma tissue. Asterisks (*) indicate skeletal muscle fibers. Scale bars, 20 μm. **e** Representative images of IF for tdTomato and PKM2 on *KP M2*^*−/−*^ sarcoma tissue

To characterize the PKM expression patterns of sarcomas arising in *KP M2*^*+/+*^ and *KP M2*^*fl/fl*^ mice, we performed Western blot analysis of PKM1 and PKM2 expression in primary tumors. While *KP M2*^*+/+*^ tumors primarily expressed PKM2, tumors from *KP M2*^*fl/fl*^ mice (*KP M2*^*−/−*^ tumors) lost PKM2 expression and expressed PKM1 (Fig. 4a). Consistent with our protein analysis, qRT-PCR analysis of the same tumors showed very low transcript levels for *Pkm2* accompanied by a dramatic increase in the transcript levels for *Pkm1* in *KP M2*^*−/−*^ tumors relative to *KP M2*^*+/+*^ tumors (Fig. 4b). Of note, the levels of *Pkm1* in *KP M2*^*−/−*^ tumors were lower than the levels of *Pkm1* in wild-type untransformed skeletal muscle tissue.

This quantitative assessment of mRNA abundance also revealed that the levels of total *Pkm* transcripts were reduced in *KP M2*^*−/−*^ tumors compared to *KP M2*^*+/+*^ tumors. The observed decrease in total *Pkm* transcripts in *KP M2*^*−/−*^ tumors was accompanied by a reduction in the maximum rate of reaction (V_max_) when pyruvate kinase enzyme activity was assessed in *KP M2*^*−/−*^ tumor lysates compared to *KP M2*^*+/+*^ tumor lysates (Fig. 4c). Interestingly, normal skeletal muscle tissue showed a significantly higher V_max_ for pyruvate kinase enzyme activity than tumors of either genotype. These findings are consistent with what has been described in adult tissue isolated from germline *Pkm2* knockout mice [2] and suggest a common mechanism of decreased total *Pkm* expression to compensate for replacement of PKM2 by the more active PKM1 isozyme.

To further characterize the PKM expression patterns of sarcomas from *KP*; *M2*^*+/+*^ and *KP*; *M2*^*fl/fl*^ mice at the cell and tissue level, we performed IHC for PKM1 and PKM2 (Fig. 4d). Consistent with Western blot analysis, *KP M2*^*+/+*^ tumors primarily and uniformly expressed PKM2. In contrast, *KP M2*^*−/−*^ tumors consisted primarily of PKM1-expressing cells and also contained a number of cells that had high expression of PKM2. To determine whether the PKM2-expressing cells in *KP M2*^*−/−*^ tumors were cancer cells, we performed immunofluorescence (IF) for PKM2 (Fig. 4e). We used *KP* mice for this study that carried a fluorescent Cre-reporter allele (*Rosa26*^*LSL-tdTomato*^), allowing us to distinguish tumor cells from stroma through tumor-specific expression of the red-fluorescent protein, tdTomato [27]. Consistent with efficient deletion of PKM2 and consequent expression of PKM1 in tumor cells, we found that PKM2 was expressed exclusively in the tdTomato-negative stromal cells. Collectively, these data argue that while loss of PKM2 concomitant with *KP* sarcoma initiation delays tumor onset, it does not fully abrogate the eventual outgrowth of tumors in most cases.

### Expression of *PKM1* in late-stage soft tissue sarcomas does not limit proliferation

**Fig. 5:**
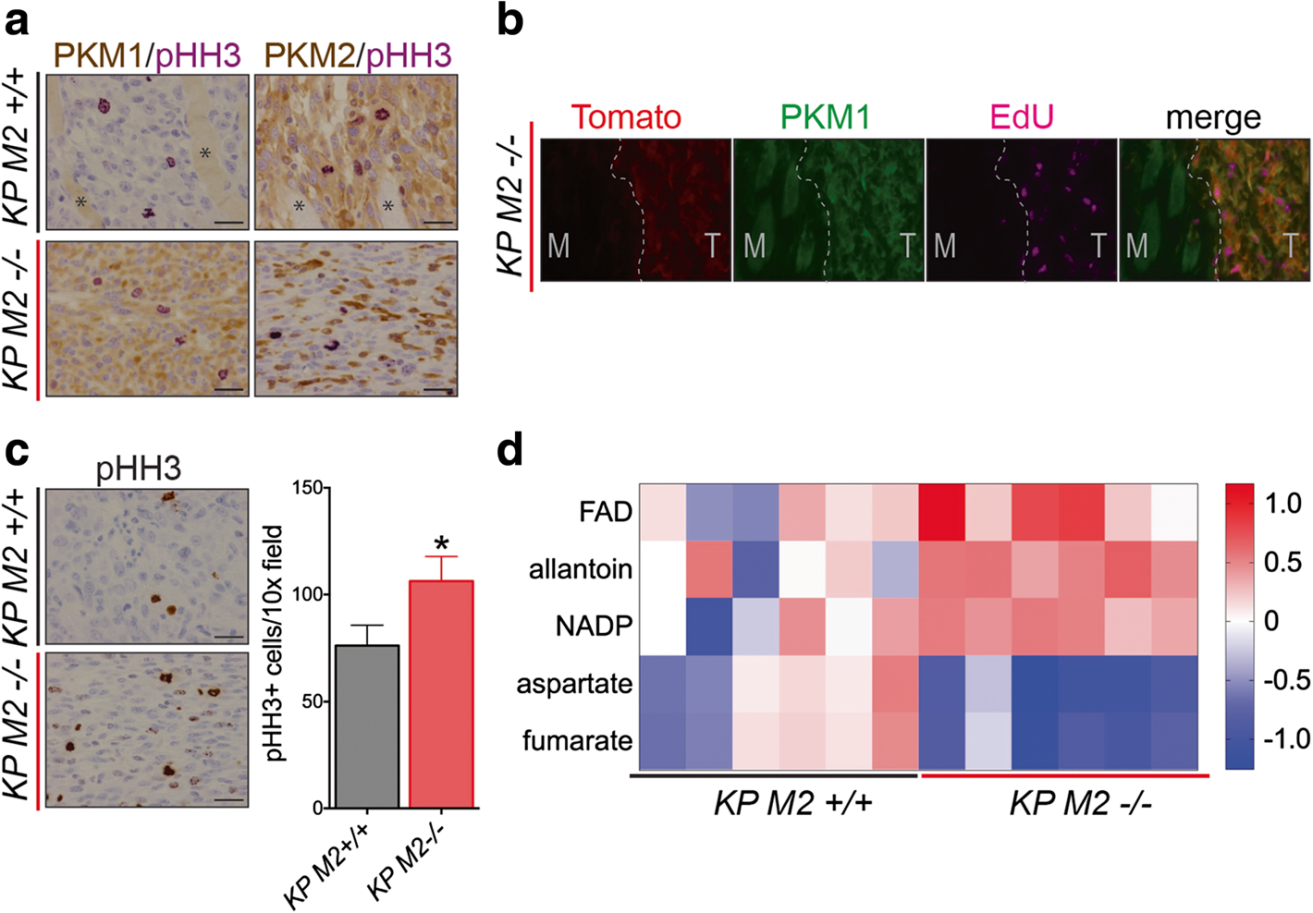
*PKM2*-deficient sarcoma tumors have increased proliferation and an altered metabolic profile. **a** Representative images of dual-color IHC for PKM1 or PKM2 (brown) and pHH3 (purple) on *KP M2*^*+/+*^ and *KP M2*^*−/−*^ sarcoma tissue. Scale bars, 20 μm. **b** Representative images of IF for tdTomato, PKM1, and EdU on *KP M2*^*−/−*^ sarcoma tissue. **c** Quantification of pHH3+ cells in *KP M2*^*+/+*^ and *KP M2*^*−/−*^ sarcomas. Representative images of the IHC for pHH3 are shown. Scale bars, 20 μm, *n* = 6 tumors per genotype. *p* value using unpaired *t* test is shown, *p < 0.05. **d** Heatmap showing measured metabolites that were significantly different between *KP M2*^*+/+*^ and *KP M2*^*−/−*^ sarcoma tissue. A full list of the measured metabolites is included in Additional file 4: Table S1

Expression of PKM1 in tumor cells has been shown to limit proliferation and progression in some mouse models of cancer [28]. Furthermore, in breast cancers with *PKM2* deletion, PKM1 expression was only observed in the non-proliferating population of tumor cells [17]. To ask whether the proliferating cells in *KP M2*^*−/−*^ sarcomas expressed PKM1, we performed two-color IHC for PKM1 or PKM2 and the proliferative marker, phospho-histone H3 (pHH3) (Fig. 5a). Contrary to the conclusions from breast cancer, we found that a large portion of PKM1-expressing tumor cells in *KP M2*^*−/−*^ sarcomas also expressed pHH3. Thus, once these tumors have been initiated, PKM1 expression does not appear to abrogate tumor cell proliferation. Analysis of 5-ethynyl-2′-deoxyuridine (EdU) incorporation in tdTomato-positive tumor cells was also consistent with this conclusion (Fig. 5b), as was an analysis of proliferating cells in cell lines derived from primary tumors in *KP M2*^*+/+*^ and *KP M2*^*fl/fl*^ mice (Additional file 3: Figure S3A). Furthermore, expression of PKM1 in sarcoma cell lines derived from *KP M2*^*−/−*^ tumors did not affect the rates of in vitro proliferation as measured through cumulative populations doublings, which were variable across cell lines but did not correlate with *Pkm* genotype (Additional file 3: Figure S3B).

Despite the longer time to palpable tumor formation and the decreased tumor penetrance in *KP M2*^*fl/fl*^ mice, there was no significant difference in the tumor weight at time of death of *KP M2*^*fl/fl*^ mice relative to *KP M2*^*+/+*^ mice (Additional file 3: Figure S3C). To determine whether *KP M2*^*−/−*^ tumors had higher rates of proliferation than *KP M2*^*+/+*^ tumors, we quantified pHH3-positive cells in tumors collected at the time of death from mice of each genotype. Unexpectedly, we found that *KP M2*^*−/−*^ tumors had more pHH3-positive cells compared to *KP M2*^*+/+*^ tumors, consistent with the notion that PKM1 expression in late-stage KP sarcomas does not abrogate tumor proliferation or progression (Fig. 5c). These findings are also consistent with PKM2 loss promoting proliferation in some other tissues [2, 17, 18].

### *PKM2* loss in soft tissue sarcomas leads to metabolic changes

The fact that deletion of *PKM2* in our KP model of soft tissue sarcoma led to delayed tumor onset and decreased penetrance but did not appear to limit proliferation or progression of late-stage tumors raised the possibility that *KP M2−/−* tumors had arisen through an adaptive process that might modulate the effects of *PKM2* loss and consequent expression of PKM1. Therefore, we hypothesized that the adaptive process that had allowed for *KP M2*^*−/−*^ tumor outgrowth might involve changes in glucose metabolism that could be apparent as changes in metabolite pool sizes. Therefore, we conducted targeted metabolomics analysis of primary tumors derived from *KP M2*^*+/+*^ and *KP M2*^*fl/fl*^ mice. The metabolite profiles of primary tumor tissue revealed significant differences in levels of five metabolites in *KP M2*^*−/−*^ tumors compared to *KP M2*^*+/+*^ tumors: FAD, NADP, and allantoin, which were increased, and aspartate and fumarate, which were decreased (Fig. 5d, Additional file 4: Table S1). Although it is unclear how these metabolic changes are related to *PKM2* loss, it is notable that allantoin is a purine catabolite and aspartate plays an important role in purine biosynthesis.

## Discussion

Here, we show that *PKM2* loss concomitant with tumor initiation leads to delayed sarcoma tumor onset, which is ultimately overcome and accompanied by changes in metabolism. The delay in tumor initiation that we observe upon loss of *PKM2* suggests that tumor initiation in this model is partially dependent on the activation of PKM2 expression in tumor-initiating cells. While the exact cell of origin for soft tissue sarcoma in this mouse model is not well defined, several studies suggest that a diverse array of skeletal muscle cell types, including mature skeletal muscle cells and skeletal muscle progenitor cells, can give rise to sarcomas with comparable frequency in this model [29, 30]. The heterogeneity in potential cells of origin for sarcomas raises the possibility that the consequences of *PKM2* loss in this cancer type are dependent on the specific cell type that gives rise to the tumor. We have found that while isolated Pax7+ and Sca1+ skeletal muscle progenitor cells express appreciable levels of *Pkm1* mRNA, they also express high levels of *Pkm2* mRNA (data not shown).

The fact that *KP M2*^*−/−*^ sarcomas were ultimately comparable in size to *KP M2*^*+/+*^ sarcomas shows that the consequences of *PKM2* loss can be overcome. Moreover, the metabolic changes that we observe in *KP M2*^*−/−*^ tumors might be indicative of a compensatory change in the metabolic program of these cells compared to *KP M2*^*+/+*^ tumor cells. It is interesting to note that while PKM1 expression in *PKM2-null* breast tumors was shown to be limited to a subpopulation of non-proliferating cells [17], PKM1 was uniformly expressed in *KP M2*^*−/−*^ sarcomas and did not abrogate proliferation in these cells. This may represent a better tolerance for PKM1 expression in non-epithelial cancers. Consistent with these in vivo data, sarcoma cell lines derived from *KP M2*^*−/−*^ tumors also expressed PKM1, and this did not affect their rates of in vitro proliferation. The fact that the in vitro proliferation of *KP M2*^*+/+*^ and *KP M2*^*−/−*^ cell lines were not significantly different even though end-stage *KP M2*^*−/−*^ tumors contained a higher number of proliferating cells than end-stage *KP M2*^*+/+*^ tumors may point to a role for the microenvironment in allowing end-stage *KP M2*^*−/−*^ tumors to catch up to their *KP M2*^*+/+*^ counterparts. Further investigation is needed to delineate the exact mechanism by which *KP M2*^*−/−*^ sarcoma tumors overcome *PKM2* loss.

This study adds to a growing body of evidence that the importance of PKM2 expression in tumor cells varies across tumor types and might be indicative of different tumor-specific metabolic requirements [16, 17, 19]. Importantly, these results have general implications for the use of therapeutic strategies that target PKM2. The success of PKM2-targeted therapies is likely to depend on both the tumor type and the stage at which a tumor is treated.

## Conclusions

Our study found that PKM2 loss at the time of tumor initiation in a mouse model of STS driven by activation of oncogenic *Kras* and loss of *p53* leads to delayed tumor onset and decreased tumor penetrance. Nonetheless, PKM2 was not required for eventual tumor formation or outgrowth. *KP M2*^*−/−*^ tumors express PKM1 and contain infiltrating stromal cells that are PKM2-positive. PKM1 expression in these tumors did not abrogate tumor cell proliferation and, in fact, end-stage *KP M2*^*−/−*^ tumors contain more cancer cells that stain positive for proliferation markers than end-stage *KP M2*^*+/+*^ tumors. We show that *KP M2*^*−/−*^ tumors have an altered metabolite profile, suggesting metabolic changes accompany loss of PKM2 expression in these tumors.

## Additional files

Additional file1: **Figure S1.** Quantification of PKM1 and PKM2 staining intensities shown as percent of tissue cores scored in 16 normal human skeletal muscle samples (A) and 48 primary human rhabdomyosarcomas (B). Score 0 = no staining, Score 1 = weak, Score 2 = positive, or Score 3 = strong. (C) Representative images of IHC for PKM1 and PKM2 in *KP* mouse sarcoma tissue. Corresponding H&E images are shown. Scale bars, 20 μm. (JPG 1314 kb)

Additional file 2: **Figure S2.** (A) Kaplan–Meier 5-year survival analysis comparing patients in the top quintile of PKM2/PKM1 expression ratio (*n* = 51; red) and all other patients combined (*n* = 208; blue). Log-rank test *p* value is shown. (JPG 683 kb)

Additional file 3: **Figure S3.** (A) Representative images of IF for tdTomato, PKM1 or PKM2, and EdU on *KP M2*^*+/+*^ or *KP M2*^*−/−*^ sarcoma cell lines. (B) Cumulative populaion doublings of *KP M2*^*+/+*^ or *KP M2*^*−/−*^ sarcoma cell lines, *n* = 3 *KP M2*^*+/+*^ cell lines and *n* = 4 *KP M2*^*−/−*^ cell lines. (C) Sarcoma weight in grams. (JPG 2694 kb)

Additional file 4: **Table S1.** Raw data for LC-MS metabolomics on KP M2+/+ and KP M2-/- sarcomas. Related to Fig. 5. This table contains all the LC-MS metabolomics data. Includes normalization and statistical analysis for each measured metabolite. (XLSX 140 kb)
