# Supplementary figures and images for: Isoform-specific deletion of PKM2 constrains tumor initiation in a mouse model of soft tissue sarcoma
Source: Cancer Metab. 2018 May 31;6:6. doi: 10.1186/s40170-018-0179-2 (PMC5977456; doi:10.1186/s40170-018-0179-2)

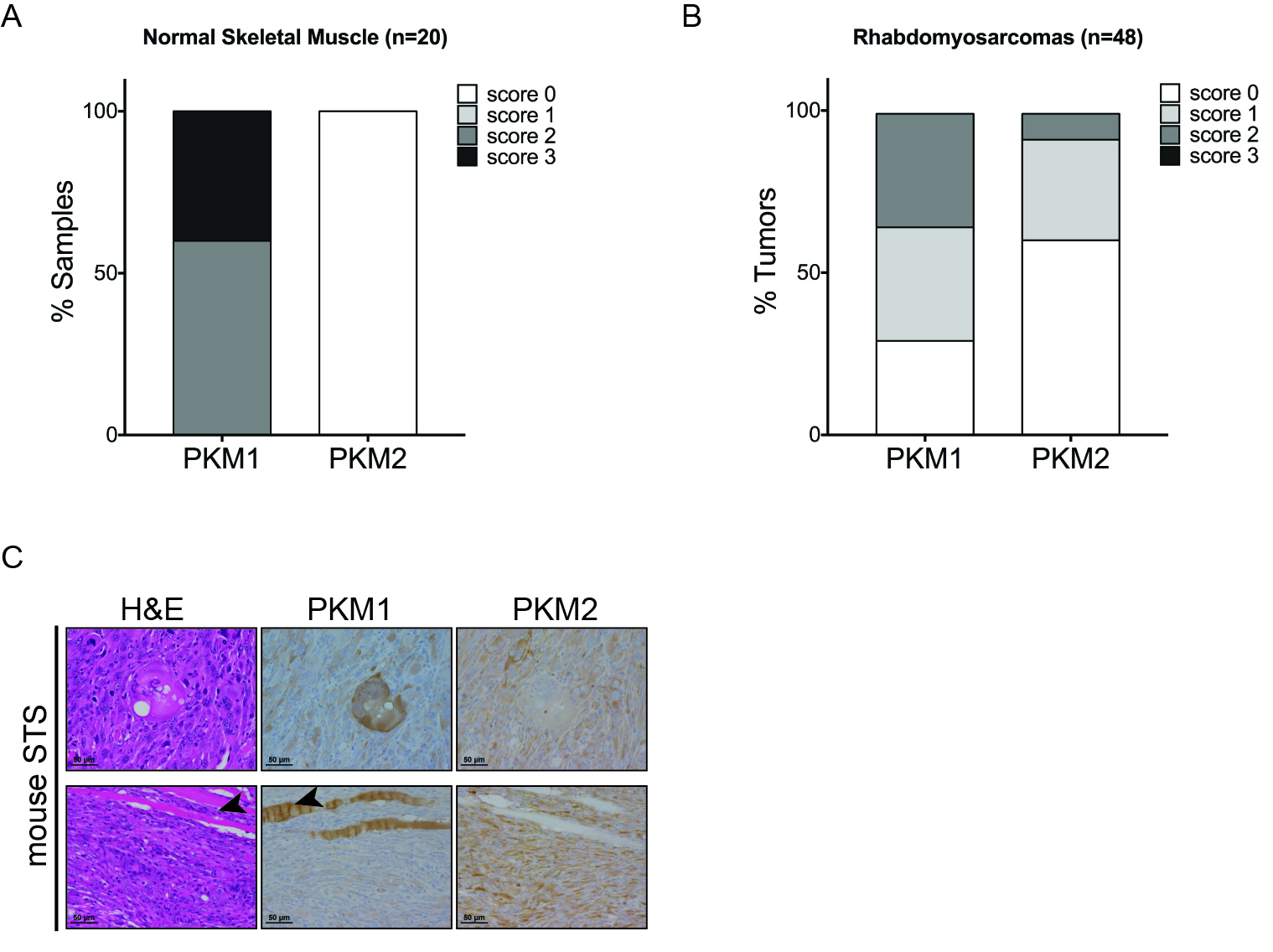

Supplement: Supplementary file 1 — Figure S1. Quantification of PKM1 and PKM2 staining intensities shown as percent of tissue cores scored in 16 normal human skeletal muscle samples (A) and 48 primary human rhabdomyosarcomas (B). Score 0 = no staining, Score 1 = weak, Score 2 = positive, or Score 3 = strong. (C) Representative images of IHC for PKM1 and PKM2 in KP mouse sarcoma tissue. Corresponding H&E images are shown. Scale bars, 20 μm. (JPG 1314 kb) [file 40170_2018_179_MOESM1_ESM.jpg]

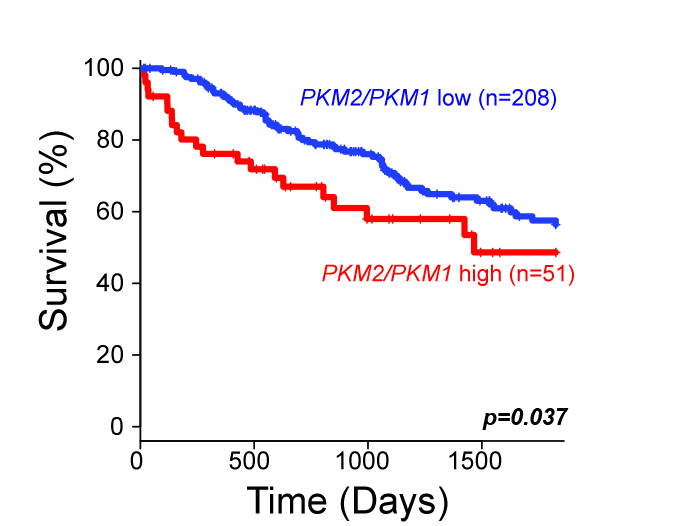

Supplement: Supplementary file 2 — Figure S2. (A) Kaplan–Meier 5-year survival analysis comparing patients in the top quintile of PKM2/PKM1 expression ratio (n = 51; red) and all other patients combined (n = 208; blue). Log-rank test p value is shown. (JPG 683 kb) [file 40170_2018_179_MOESM2_ESM.jpg]

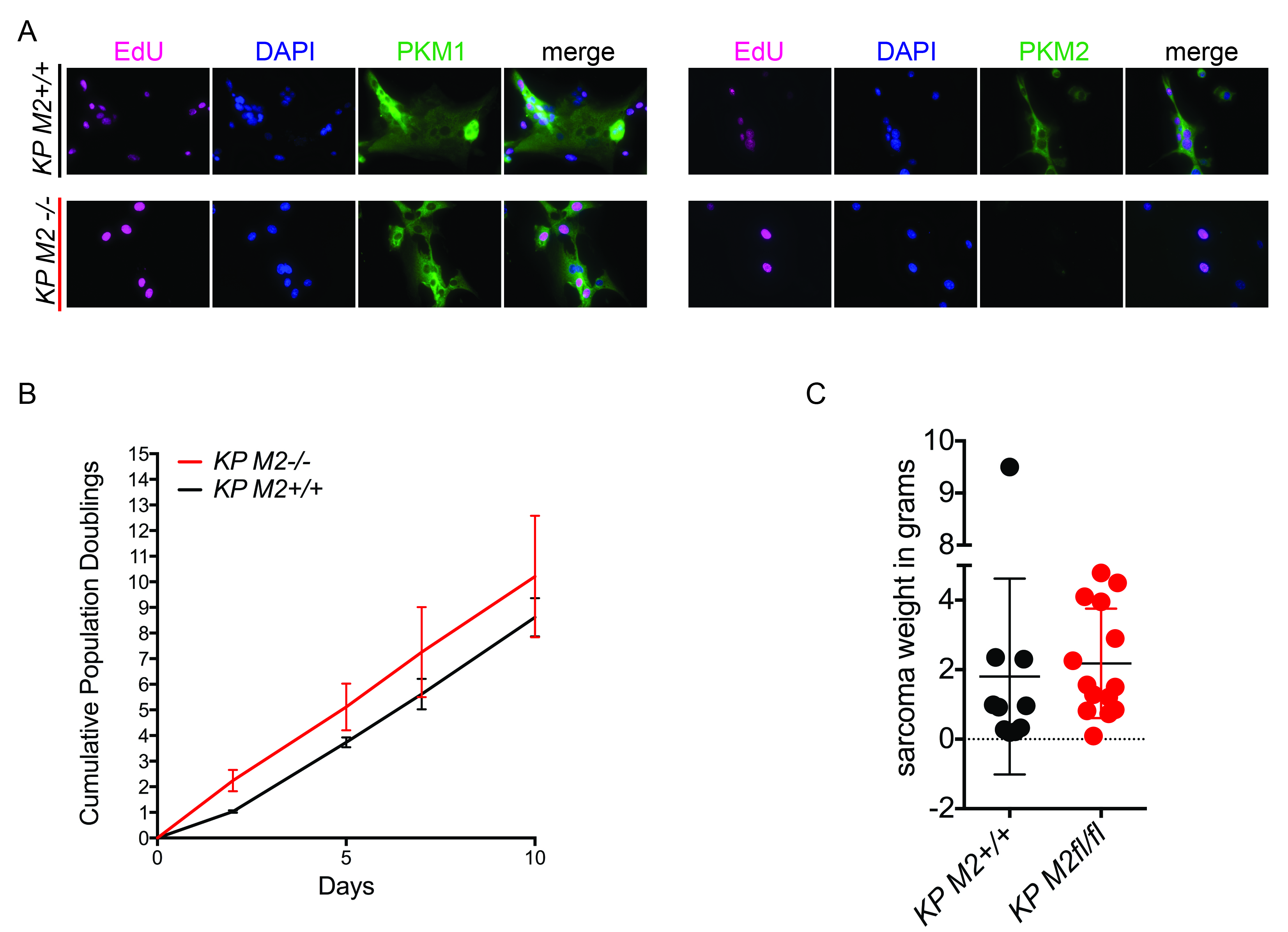

Supplement: Supplementary file 3 — Figure S3. (A) Representative images of IF for tdTomato, PKM1 or PKM2, and EdU on KP M2+/+ or KP M2−/− sarcoma cell lines. (B) Cumulative populaion doublings of KP M2+/+ or KP M2−/− sarcoma cell lines, n = 3 KP M2+/+ cell lines and n = 4 KP M2−/− cell lines. (C) Sarcoma weight in grams. (JPG 2694 kb) [file 40170_2018_179_MOESM3_ESM.jpg]
